# World’s Congress Notes

**Published:** 1893-01

**Authors:** 


### WORLD'S CONGRESS NOTES.

As some of the profession may not fully understand the authority of the Congress, the following extracts from public documents will make the matter plain.

"DEPARTMENT OF STATE,
"WASHINGTON, May 23d, 1892.

"One of the accompaniments with the President's invitation to the several foreign governments, issued in accordance with the Act approved April 25th, 1890, was the World's Congress Auxiliary to the World's Columbian Exposition.

"The purpose of its organization was fully stated, and among them it was proposed that a series of World's Congresses, to promote the objects in view was to be held in connection with the World's Columbian Exposition in 1893.

"'The World's Congress Auxiliary,' it added, 'has been duly authorized and organized to promote the holding and success of such Congresses.'

"I observe, in conclusion, that a representative of the World's Congress Auxiliary, a few days ago, called at the department to learn whether it would be possible to send their pamphlets to all foreign governments, with a suitable instruction to our minister to present them to the governments to which they were respectively accredited, as supplementary to the original invitation. Assurance was given that the department would gladly do so upon the receipt of a formal written request to that effect.

"I have the honor to be, sir, your obedient servant,

"JAMES G. BLAINE."

HON. JOHN SHERMAN,
*Chairman Committee on Foreign Relations,*
*United States Senate.*

THE OFFICIAL INVITATION TO FOREIGN GOVERNMENTS TO APPOINT DELEGATES TO ALL OR ANY OF THE WORLD'S CONGRESSES TO BE HELD AT CHICAGO IN 1893.

### CIRCULAR.

"DEPARTMENT OF STATE,
"WASHINGTON, June 13th, 1892.

"*To the Diplomatic and Consular Officers of the United States.*

"GENTLEMEN:—The department is in receipt of a letter from Mr. Charles C. Bonney, President of the World's Congress Auxiliary, dated Chicago, the 3d inst. It states that in pursuance of the course indicated in the original announcement of the World's Congress Auxiliary, which was transmitted with the Act of Congress approved April 25th, 1890, and the President's invitation of January 14th, 1891, extending to all foreign governments a cordial invitation to participate in the World's Columbian Exposition to be held in Chicago in 1893, the work of the World's Congress Auxiliary has been organized.

"It is particularly requested that a convenient number of the most eminent representatives of the various departments of human progress be selected as delegates to attend the respective Congresses. On receipt of the names of such delegates suitable communications will be promptly forwarded to them.

"I am, gentlemen, your obedient servant,

"WILLIAM F. WHARTON,

"*Acting Secretary.*"

Under this authority Hon. C. C. Bonney, President of the World's Congress Auxiliary, appointed:

J. S. MITCHELL, M. D., *Chairman*,
R. LUDLAM, M. D., *Vice-Chairman*,
*Committee on a Congress of Homœopathic Physicians and Surgeons.*

JULIA HOLMES SMITH, M. D., *Chairman*,
ELIZABETH McCRACKEN, *Vice-Chairman*,
*Woman's Committee on a Congress of Homœopathic Physicians and Surgeons.*

### WORLD'S CONGRESS NOTES.

P. C. Majumdar, L. M. S., of Calcutta, India, editor of the *India Homœopathic Review*, who wrote the history of Homœopathy in India for the Atlantic City Congress, will personally attend the Chicago Congress, and hopes to be able to give "a very cheerful account of the progress and advancement of Homœopathy in India."

Dr. E. T. Adams, a prominent member of our school in Toronto, Canada, will attend the Congress, and is taking an active interest in its success.

Dr. B. N. Banerjee, who also sent a very interesting account of Homœopathy in India to the last Congress, writes that he will be present at the World's Congress in Chicago. Both Dr.

Majumdar and Dr. Banerjee are good English scholars, and will add greatly to the interest of the sessions.

Engagements for rooms at the hotel already made indicate that the profession will be well represented at the Congress. Rooms will be furnished during the week of the Congress at regular rates.

Address, Great Northern Hotel, Chicago, Illinois.

